# Statins but not fibrates improve the atherogenic to anti-atherogenic lipoprotein particle ratio: a randomized crossover study

**DOI:** 10.1186/1472-6904-8-10

**Published:** 2008-10-28

**Authors:** Sammy Y Chan, GB John Mancini, Andrew Ignaszewski, Jiri Frohlich

**Affiliations:** 1 Division of Cardiology, Department of Medicine, University of British Columbia, Vancouver, Canada; 2 Healthy Heart Program, St. Paul's Hospital, Vancouver, Canada; 3 Department of Laboratory Medicine, University of British Columbia, Vancouver, Canada

## Abstract

**Background:**

Prior studies suggested low density lipoprotein particle (LDLP) size is a predictor of atherosclerosis. Knowledge of effects of lipid lowering drugs on lipoprotein subclasses is useful. We treated subjects with hyperlipidemia sequentially with statins and fibrates, the 2 main classes of lipid lowering therapy and studied changes in NMR lipoprotein subclasses.

**Methods:**

35 subjects (21 males; 60 ± 12 y) were enrolled in a crossover study. Subjects had baseline lipid profile & apoB. Lipoprotein subclasses, particle numbers and diameters were assessed with NMR spectroscopy. Subjects were randomized to simvastatin 20 mg or fenofibrate 200 mg. Repeat testing was done at 12 weeks. After 6 week washout, subjects were started on alternate drug for 12 weeks with pre/post tests.

**Results:**

Both therapies resulted in expected changes in lipids and apoB. Decreases in total cholesterol, LDL and apoB were greater with simvastatin. Fenofibrate led to small increase in HDL. Both therapies decreased LDLP. Reduction in LDLP was greater with simvastatin (32%, p < .001) compared to fenofibrate (17%; p = .036 vs pre; p = .027 vs simvastatin end). Fenofibrate resulted in 17% rise in large LDLP (p = .06 vs pre) and 32% drop in small LDLP (p = .007 vs pre). Simvastatin led to decrease in both LDLP fractions (19% large LDLP; p = .001 vs fenofibrate end; 34% small LDLP, p = .019 vs pre). With fenofibrate, LDLP size increased from 20.4 nm to 20.8 nm (p = .037). There was no change in LDLP size with simvastatin. There was 18% increase in HDL particle number (HDLP) with fenofibrate (p = .05). There were no changes in HDLP with simvastatin. There were no changes in HDLP size with either drug. Pre- and post-therapy LDLP/HDLP ratio was similar with fenofibrate but was reduced by simvastatin (p = .045).

**Conclusion:**

Simvastatin reduced LDLP across all subclasses with no effect on size. Simvastatin had no effect on HDLP. Fenofibrate had weak effect on LDLP number but increased LDLP size by raising large LDLP and reducing small LDLP. Fenofibrate had weak effect on HDLP number with no change in size. Importantly, net atherogenic to antiatherogenic lipoprotein ratio (LDLP/HDLP) was reduced by simvastatin but not by fenofibrate.

## Background

Prospective, placebo controlled primary and secondary prevention trials have shown that cholesterol lowering reduces cardiovascular morbidity and mortality[1,2]. Most of the emphasis has been placed on lowering of low density lipoprotein (LDL) cholesterol. Although it is usually thought of as a single entity, LDL is actually comprised of multiple different subclasses that differ in size, density, physicochemical composition, buoyancy and metabolic behaviour. All these factors influence their atherogenicity[3,4]. In a detailed analysis of the factors associated with angiographic progression/regression in the Familial Atherosclerosis Treatment Study, Zambon et al showed that LDL buoyancy accounted for 37% of the variance whereas apolipoprotein B (a measure of number of LDL particles) only accounted for 12%[5]. Furthermore, different LDL profiles may warrant different therapies. The same investigators showed that subjects with predominantly large and buoyant LDL require aggressive reduction of LDL cholesterol whereas those with Familial Combined Hypercholesterolemia require LDL cholesterol lowering as well as reduction in the number of small, dense LDL particles[6].

Different techniques can be used to quantify LDL into different classes. These include fast protein liquid chromatography, nuclear magnetic resonance (NMR) spectroscopy, disc polyacrylamide electrophoresis, density gradient ultracentrifugation and gradient gel electrophoresis[7]. Among these, NMR spectroscopy has the advantage of being able to measure simultaneously particle concentration, lipid mass-weighted particle diameter and the predominant class of both LDL and high density lipoprotein (HDL)[8].

3-hydroxy-3-methylglutaryl-coenzyme A (HMG Co-A) reductase inhibitors (statins) and fibric acid derivatives (fibrates) are two widely used agents for treatment of dyslipidemia[1,9]. Statins have been shown to improve cardiovascular mortality and outcomes in multiple clinical trials. Detailed analyses of angiographic trials of statin therapy have yielded important insights. In patients with CAD and "normal" cholesterol, the progression of disease as defined by progressive angiographic narrowing has been shown to correlate with concentration of intermediate density lipoprotein (IDL) cholesterol and HDL cholesterol but not with LDL cholesterol[10]. The NHLBI-II trial reported that subjects with greater angiographic "stability" had significantly greater reductions in total LDL mass, small dense LDL and IDL mass[11]. Among the successfully treated subjects in the MARS trial, triglyceride rich lipoprotein levels were the predominant predictors of progression[12]. In the STARS trial, reduction in small, dense LDL was the best predictor of angiographic improvement[13]. In a detailed analysis, investigators from the Stanford Coronary Risk Intervention Project showed that despite almost identical LDL cholesterol at baseline and comparable diet and drug induced reduction in levels of LDL cholesterol, patients with subclass pattern B (predominantly small dense LDL), but not those with pattern A (predominantly large, buoyant LDL) showed progression of atherosclerosis[14]. These studies illustrate the importance of LDL particle size to progression of atherosclerosis and adverse cardiac outcomes.

Fibrates are another major class of lipid modifying drug. Clinical trials of fibrates have revealed conflicting results. Of the 5 major fibrate trials in the modern era, only the VA-HIT study with gemfibrozil showed a significant reduction in major cardiovascular events as well as stroke[9]. Detailed analysis of the lipid profile in the VA-HIT trial showed that both LDL particle numbers and HDL particle numbers were independent predictors of adverse outcome whereas LDL cholesterol and HDL cholesterol were not[15].

Knowledge of the effects of different classes of lipid lowering agents on lipoprotein subclasses, particularly on the particle concentration and size, is essential. In this study we treated subjects with hypercholesterolemia sequentially with statins and fibrates, the two main classes of lipid lowering drugs and studied the changes in lipoprotein subclasses and average particle diameters with an NMR method.

## Methods

Thirty-five subjects (21 males (60%), mean age 60 ± 12 years (median 59 years)). were enrolled. Subjects were recruited from a Lipid Clinic at a tertiary care referral hospital. None had coronary artery disease or other vascular disease by history. All participants underwent baseline clinical and laboratory assessment including lipid profile, apoB, lipoprotein subclasses, particle numbers and average particle diameters, high sensitivity C-reactive protein, glucose, creatinine and homocysteine. Subjects were then randomized in a 1:1 fashion to either initial therapy with simvastatin 20 mg daily or fenofibrate (lipidil micro) 200 mg daily for 12 weeks. Repeat final laboratory assessment was carried out at 12 weeks. After a washout period of 6 weeks, subjects were started on the alternate drug for 12 weeks with pre and post laboratory testing. A lipid profile was carried out after 6 weeks in the simvastatin group. If LDL-C was ≥ 2.5 mmol/L, the simvastatin dose was increased to 40 mg daily. This occurred in 3 subjects. The study was approved by the Providence Healthcare/University of British Columbia ethics review board. All subjects gave written informed consent.

Lipoprotein lipids, Apo B, homocysteine, glucose were measured by standard clinical methods in a clinical laboratory (St. Paul's Hospital, Vancouver, Canada). C-reactive protein was measured with a high sensitivity immunoassay (Immulite 2000, Siemens Medical Solutions Diagnostics, Tarrytown, New York). Lipid subclasses, particle numbers and average particle diameters were measured by NMR spectroscopy (Liposcience Inc, Raleigh, North Carolina). Details of NMR analyses have been published previously[15]. In brief, the particle concentrations of lipoprotein subclasses of different sizes are derived from the measured amplitudes of the distinct lipid methyl group NMR signals they emit. For simplicity of analysis very low density lipoprotein particles (VLDL-P) were divided into large VLDL-P (60–200 nm), medium VLDL-P (35–60 nm) and small VLDL-P (27–35 nm). LDL particle (LDL-P) subclasses were divided into intermediate density lipoprotein-particles (23–27 nm), large LDL-P (21.2–23.0 nm) and small LDL-P (18.0–21.2 nm). HDL particle (HDL-P) subclasses were divided into large HDL-P (8.8–13.0 nm), medium HDL-P (8.2–8.8 nm) and small HDL-P (7.3–8.2 nm). These subgroups are consistent with previous studies using NMR spectroscopy. The lipid mass-weighted particle diameters of VLDL, LDL and HDL were also obtained with NMR spectroscopy. The proportions of subjects with predominantly large LDL (average particle diameter >21.2 nm) and small LDL (average particle diameter ≤ 21.2 nm) were determined.

### Statistical analysis

There were no significant differences in any of the lipid variables at either the initiation or the end of treatment for either drug regardless of which drug was used first. Therefore the data were combined into the 2 drug treatment groups for final analyses. Data was visually inspected for normality. Data with obvious skewness were log transformed. Results were expressed as mean ± SD or median with interquartile range where appropriate. The primary outcomes were comparison of the change from baseline to end of 12 weeks between simvastatin and fenofibrate. Student t-test or Mann-Whitney U test was used for comparison between variables. A p-value of <0.05 was considered significant for the comparisons.

## Results

### Subject characteristics

The risk factor profile, laboratory parameters and baseline lipid profiles were listed in Table 1. Subjects were at relatively low risk for cardiovascular events. There were no significant changes in glucose, insulin, creatinine, fibrinogen, C-reactive protein with either simvastatin or fenofibrate treatment.

**Table 1:** Baseline characteristics of the subjects

Glucose (mmol/L)	5.5 ± 1.2
Insulin (pmol/L)	55.5 ± 46.9
HOMA	302 ± 259
Creatinine (mmol/L)	85 ± 17
C reactive protein (mg/dl)	2.7 ± 3.9
Fibrinogen (g/L)	3.3 ± 0.6
Weight (kg)	82.1 ± 12.8
Height (cm)	170.2 ± 9.8
Body mass index	28.5 ± 4.2
Lipid profile	
Total Cholesterol (mmol/L)	6.7 ± 1.3
LDL-C (mmol/L)	4.4 ± 1.1
HDL-C (mmol/L)	1.1 ± 0.4
Triglycerides (mmol/L)	2.6 ± 1.4
ApoB (g/L)	1.4 ± 0.3

### Lipid profile

**Figure 1:**
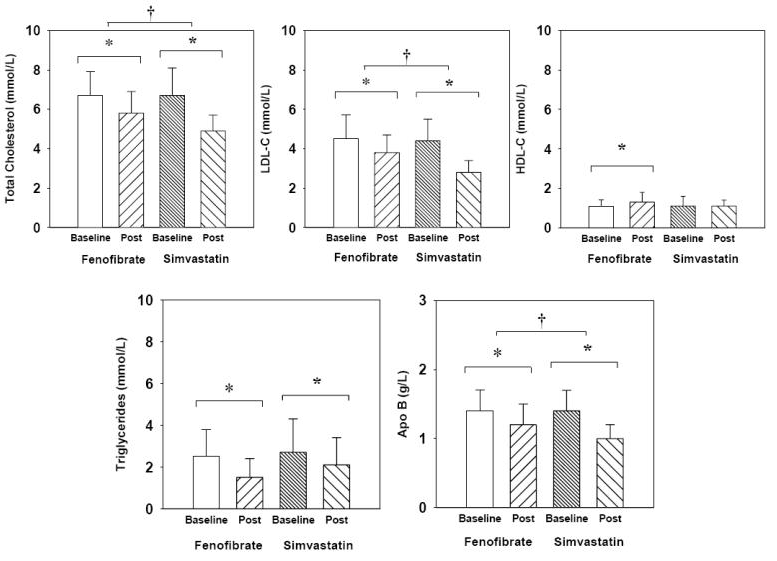
**Changes in lipid profile with simvastatin and fenofibrate therapy**. All lipid parameters including apoB improve significantly with both simvastatin and fenofibrate treatment. However, the reduction in total cholesterol, LDL-C and apoB (as indicated by the uppermost bar) were significantly higher with simvastatin than fenofibrate. * p < 0.05 vs baseline; † p < 0.05 vs other drug.

At baseline, all subjects had relatively high total cholesterol, LDL-C and apoB. Both fenofibrate and simvastatin therapy resulted in significant changes in all parameters of the lipid profile as well as apoB (Figure 1). However, the decreases in total cholesterol, LDL-C and apoB were significantly greater with simvastatin therapy. Fenofibrate therapy led to a marginally higher increase in HDL cholesterol only.

### NMR lipoprotein particle numbers and sizes

**Figure 2:**
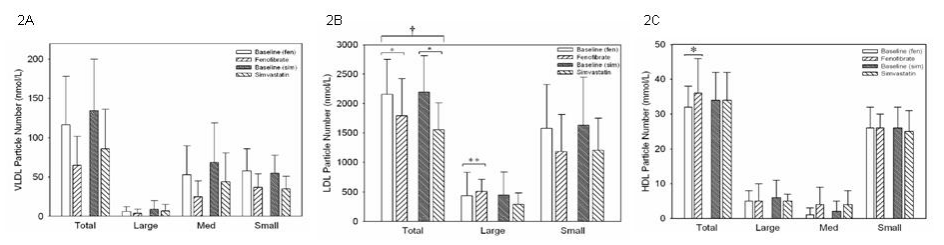
**Changes in particle numbers of lipid subfractions with simvastatin and fenofibrate therapy**. A: VLDL subfractions – no significant changes were seen in VLDL particle number with either simvastatin or fenofibrate therapy. B: LDL subfractions – both simvastatin and fenofibrate reduce total LDL particle numbers. The reduction was higher with simvastatin than fenofibrate (as illustrated by the uppermost bar). There was a significant increase in the large LDL-P subfraction with fenofibrate. Small LDL particle numbers tend to reduce with both therapies. C: HDL subfractions – fenofibrate but not simvastatin increase total HDL particle numbers. * p < 0.05 vs baseline; ** p < 0.05 vs baseline; † p < 0.05 vs other drug.

**Figure 3:**
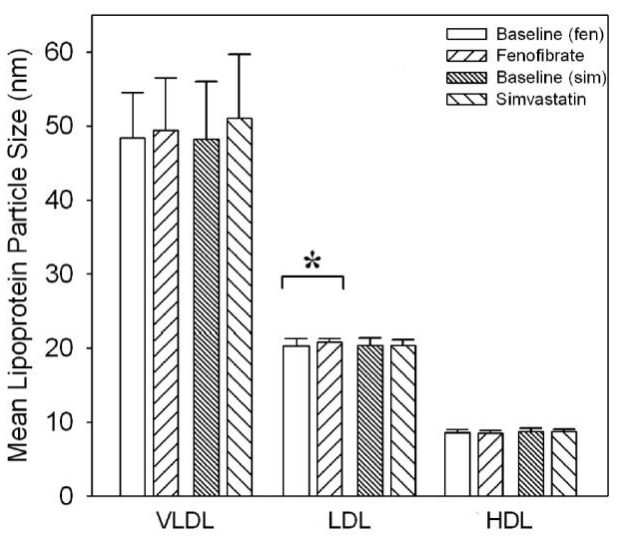
**Changes in mean particle diameters of lipid subfractions with simvastatin and fenofibrate therapy**. Fenofibrate but not simvastatin therapy significantly increase mean LDL particle diameter. There were no changes with either drugs on mean VLDL or HDL particle diameters. * p < 0.05 vs baseline.

At baseline, the majority of VLDL-P were in the medium (45% before fenofibrate therapy, 52% before simvastatin therapy) and small subfractions (49% before fenofibrate therapy, 41% before simvastatin therapy) (figure 2A). Both fenofibrate and simvastatin reduced total VLDL-P with only minor differences between the two therapies (50% after fenofibrate therapy, 39% after simvastatin therapy). All subclasses of VLDL-P were reduced with both fenofibrate and simvastatin therapy with no difference between the two drugs. There were no changes in VLDL-P diameter with either therapy (figure 3). It should be noted that VLDL particles degrade during freeze thaw cycles. As our samples were frozen prior to being thawed and analyzed for different lengths of time, we cannot be certain of any changes observed in our subjects with either of the drugs. We have included the VLDL particle results for completeness only.

Almost all subjects had a predominance of small dense LDL-P at baseline. Fibrate therapy resulted in no change (88% to 86%). With simvastatin therapy, all subjects had predominance of small dense LDL-P (from 96% at baseline to 100% with treatment). Both fenofibrate and simvastatin decreased total LDL-P concentration (figure 2B). With fenofibrate, there was a significant 21% decrease in LDL-P concentration (from 2156 ± 596 nmol/L to 1794 ± 634 nmol/L, p = 0.036). With simvastatin, LDL-P decreased by 32% (from 2194 ± 619 nmol/L to 1553 ± 461 nmol/L, p < 0.001). The reduction in LDL-P was significantly greater in the simvastatin arm compared to the fenofibrate arm (p = 0.027). Fibrate therapy resulted in a significant 17% increase in the large LDL-P fraction but a 32% decrease in the small LDL-P fraction. Simvastatin therapy led to a decrease in both LDL-P fractions (-19% in large LDL-P; -34% in small LDL-P). At the end of therapy, 66% of the LDL-P was in the small subfraction after fenofibrate therapy while 77% of the LDL-P was in the small subfraction after simvastatin therapy.

Before fenofibrate therapy, the mean LDL particle size at baseline was 20.4 ± 1.0 nm (figure 3). At completion of therapy, the mean LDL particle size increased to 20.8 ± 0.5 nm (p = 0.037). Before simvastatin treatment, the mean LDL particle size at baseline was 20.2 ± 0.8 nm (p = NS vs fenofibrate baseline), at completion, the mean LDL particle size was unchanged at 20.4 ± 0.8 nm (p = NS vs baseline).

There was an increase of 18% in total HDL particle number with fenofibrate (31.9 ± 6.3 nmol/L vs 36.3 ± 9.9 nmol/L; p = 0.05, figure 2C) primarily due to an increase in the medium HDL-C fraction. There were no changes in total HDL particle number with simvastatin (33.5 ± 7.8 nmol/L vs 34.0 ± 7.9 nmol/L; p = NS). There were no changes in HDL particle size with either fenofibrate or simvastatin (figure 3).

### Atherogenic to anti-atherogenic lipoprotein particle ratio

**Figure 4:**
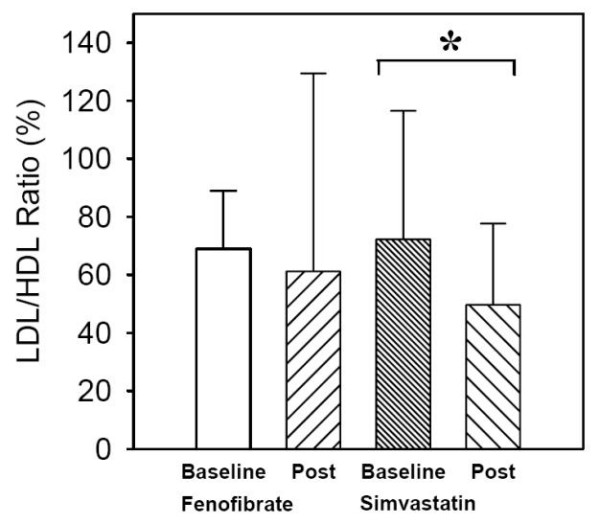
**Changes in atherogenic to anti-atherogenic particle ratio (LDL-P/HDL-P)**. Simvastatin but not fenofibrate reduce the LDL-P/HDL-P ratio significantly. * p < 0.05 vs baseline.

The pre- and post-treatment LDL-P to HDL-P ratio was similar with fenofibrate therapy (68.9 ± 20.0 vs 61.2 ± 68.0 p = NS, figure 4). However, the pre- and post-treatment LDL-P to HDL-P ratio was significantly reduced by simvastatin (72.3 ± 44.2 vs 49.7 ± 28.2 p = 0.045, figure 4).

## Discussion

Our study of dyslipidemia subjects demonstrated that simvastatin and fenofibrate modify lipid profile in different manners. Simvastatin predominantly reduced LDL-P concentration across all LDL subclasses with no change in particle diameter. Simvastatin had no effect on either HDL-P concentration or diameter. Fenofibrate had a much weaker effect on LDL-P concentration but increased LDL-P diameter; it also had a moderate effect on increasing relative HDL-P concentration but did not change the HDL-P diameter. Importantly, the net overall atherogenic to anti-atherogenic lipoprotein ratio (as reflected by the LDL-P:HDL-P ratio) was significantly reduced by simvastatin but was not affected by fenofibrate. This finding has not been reported before.

Our results are consistent with previous investigations of fenofibrate therapy using NMR spectroscopy. Ikewaki et al studied 20 subjects with hypertriglyceridemia[16]. They found a 97% increase in large LDL-P and 42% decrease in small LDL-P while the overall LDL-P particle number was only decreased by 10% and the LDL-P diameter increased. There were decreases in large HDL-P and increases in small and medium HDL-P with a significant increase in overall HDL-P number (27%) and HDL-P diameter decreased. These same investigators found similar changes in a subsequent study with bezafibrate suggesting the changes in LDL-P and HDL-P subclasses distribution, concentration and diameter were not drug specific but related to the effects of this drug class[17]. Furthermore, May et al found a decrease in LDL pattern B and an increase in buoyant (large) LDL particles in diabetic subjects with mixed dyslipidemia treated with fenofibrate[18]. Both HDL_2 _and HDL_3 _particles increase with fenofibrate.

Simvastatin therapy significantly improved the lipid profile. Detailed analyses of the lipid subclasses showed reductions in both the large and small LDL subfractions. There were no changes in average LDL particle size. Simvastatin therapy did not alter HDL particle number or particle size. Two previous studies had evaluated the effects of simvastatin on lipoprotein subfractions. Milller et al found a dose dependent reduction in LDL-P, IDL-P and VLDL-P as well as a dose dependent increase in large HDL-P[19]. May et al found a decrease in both LDL pattern B and buoyant LDL particles with simvastatin[18]. There were no significant changes with HDL_2 _and HDL_3 _particles. Other investigators have studied changes in lipid subfractions with other statins. Soedamah-Muthu et al studied 122 subjects with type 2 diabetes and dyslipidemia[20]. They compared changes in lipid subfractions between atorvastatin with placebo. Their findings in the atorvastatin arm are similar to our results with simvastatin.

Three previous studies have compared statin with fibrates in the same group of subjects. Melonovsky et al compared 29 subjects with combined hyperlipidemia between atorvastatin and fenofibrate[21]. They measured lipid profile, apoB, apoA1 and LDL particle size with gradient gel electrophoresis. Their findings are similar to ours. Frost et al compared 13 subjects with type 2 diabetes mellitus and mixed hyperlipidemia with atorvastatin and fenofibrate[22]. LDL particle size was determined by isopycknic density gradient ultracentrifugation. They found that atorvastatin reduced all LDL subclasses whereas fenofibrate shifted LDL subclasses from small, dense LDL (-31%) to intermediate dense LDL (+36%). Winkler et al studied 6 males with combined hyperlipidemia with atorvastatin and fenofibrate[23]. LDL particle was fractionated with ultracentrifugation. Treatment with fenofibrate resulted in increases in the larger LDL-1, LDL-2 and LDL-3 fractions and decreases in the more dense LDL-4, LDL-5 and LDL-6 fractions. Treatment with atorvastatin resulted in decreases in all 6 LDL fractions. Our study is larger than the previous studies. Furthermore, by using the NMR spectroscopy technology, we were able to provide more details with respect to changes in not only LDL subclasses, but also HDL subclasses as well as changes in LDL-P to HDL-P ratio.

Fibrates induce a moderate reduction in LDL-cholesterol with an increase in LDL particle size. The way fenofibrate increases LDL particle size is of interest. Possible mechanisms include preferential decrease in small LDL-P numbers, preferential increase in large LDL-P numbers or a combination of both. Our results suggest both an increase in large LDL-P numbers and a decrease in small LDL-P numbers.

LDL particle size has been proposed as a marker of increased risk of future adverse cardiac events. However, the utility of LDL particle size as a predictor can be questioned. Almost all of the studies that implicate LDL particle size as an independent variable for either the presence or progression of atherosclerosis have not taken LDL particle number into account. The VA-HIT study evaluated both simultaneously with combined cardiac death or nonfatal myocardial infarction as an end point[15]. LDL particle size did not emerge as an independent predictor but LDL particle number was a predictor of adverse outcomes. Similarly, HDL particle size did not predict outcome but total HDL particle number was a predictor. The EPIC-Norfolk study evaluated LDL particle number and size in apparently healthy males and females[24]. They showed that LDL particle number is a predictor of development of coronary artery disease but LDL particle size is not related to outcome. We showed that simvastatin but not fenofibrate significantly alters the atherogenic to anti-atherogenic lipoprotein LDL-P/HDL-P ratio. This fits with the results of previous studies that showed that statins improve cardiovascular outcomes while the results with fibrates as a class are, at best, mixed.

In fact, our results and other studies call into question the utility of fibrates as a therapeutic class of LDL modifying agents. Although current thought suggests that large LDL particles are less atherogenic than small LDL particles, recent findings have shown that both large and small LDL particles are atherogenic. In the VA-HIT study, the hazard ratio of large LDL particles is similar to that of small LDL particles[15]. In the Multi-Ethnic Study of Atherosclerosis, both large and small LDL particles were independently associated with carotid intima media thickness (IMT) [25]. Furthermore, on a per particle basis, large LDL particles were associated with a greater difference in IMT than small LDL particles. We found that while both fenofibrate and simvastatin lowered small LDL particles to a similar degree, fenofibrate actually increased the number of large LDL particles whereas simvastatin decreased the number of large LDL particles. The increase in large LDL particles is partially responsible for the failure of fenofibrate to alter the LDL-P/HDL-P ratio. The VA-HIT study also showed that Gemfibrozil increased the number of large LDL particles. Thus we believe that fibrates as a class increase large LDL-P and do not lower LDL-P/HDL-P ratio. These data would argue that fibrates have only a very limited role (if any at all) in modifying LDL-P and LDL cholesterol.

There are a number of limitations to this study. The number of subjects is relatively small and small differences will not be detectable. We also used a cross over design with all subjects receiving both therapies. This allowed us to determine the effects of both therapies on all subjects. There was a long washout period between the two therapies. There were no significant differences in baseline lipid profile or lipoprotein subclasses at the initiations of either the first or second drug. Nonetheless, we cannot completely exclude a carryover effect from the first to the second drug. The two therapies were applied in different manners in this study. With simvastatin, we treated to a LDL-cholesterol target (2.5 mmol/L) and thus different subjects received different doses of the drug. With fenofibrate, the same dose was applied to all subjects. However, this reflects how these therapies are used in current practice.

## Conclusion

We assessed the effects of both simvastatin and fenofibrate therapy in a group of subjects with dyslipidemia. We found that both therapies are associated with significant changes in lipid profile. Simvastatin reduced LDL cholesterol and apoB while fenofibrate increased HDL cholesterol. The effects of simvastatin and fenofibrate on LDL particle subfractions were different. Specifically, simvastatin reduced the total number of LDL particles by reducing both large and small LDL particles. Fenofibrate reduced the number of small LDL particles but increased the number of large LDL particles. The overall LDL-P/HDL-P ratio was reduced by simvastatin but not changed with fenofibrate therapy. Our results combined with previous reports call into question the overall role of fibrates in modifying LDL-P, LDL cholesterol and atherosclerosis.

## Competing interests

The authors declare that they have no competing interests.

## Authors' contributions

SYC conceived the study, designed the protocol, recruited the subjects, collected and analyzed the data, performed the statistical analyses and wrote the manuscript. GBJM designed the protocol, recruited the subjects and reviewed the manuscript. AI designed the protocol, recruited the subjects and reviewed the manuscript. JF design the protocol, recruited the subjects and reviewed the manuscript. All authors have read and approved the final manuscripts.

## Pre-publication history

The pre-publication history for this paper can be accessed here:


